# Supplementary material for: Machine Learning Accelerates Crystallization for Structure Determination
Source: Angew Chem Int Ed Engl. 2026 May 4;65(25):e1218503. doi: 10.1002/anie.1218503 (PMC13266958; doi:10.1002/anie.1218503)

checkCIF/PLATON report

Structure factors have been supplied for datablock(s) lcz003

THIS REPORT IS FOR GUIDANCE ONLY. IF USED AS PART OF A REVIEW PROCEDURE FOR PUBLICATION, IT SHOULD NOT REPLACE THE EXPERTISE OF AN EXPERIENCED CRYSTALLOGRAPHIC REFEREE.

No syntax errors found. CIF dictionary Interpreting this report

Datablock: lcz003

```
Bond precision:   C-C = 0.0168 A          Wavelength=1.54184

Cell:             a=11.5217(3)       b=12.1025(2)      c=22.8839(4)
                  alpha=91.076(2)    beta=92.848(2)    gamma=117.691(2)
Temperature:      100 K
```

	Calculated	Reported
Volume	2818.84(11)	2818.84(11)
Space group	P -1	P -1
Hall group	-P 1	-P 1
Moiety formula	C38 H14 Ag6 F36 N12 O4 [+ solvent]	2(C15 H3 Ag3 F18 N6), C8 H8 O4
Sum formula	C38 H14 Ag6 F36 N12 O4 [+ solvent]	C38 H14 Ag6 F36 N12 O4
Mr	2033.83	2033.83
Dx,g cm-3	2.396	2.396
Z	2	2
Mu (mm-1)	18.011	18.011
F000	1928.0	1928.0
F000’	1937.89	
h,k,lmax	14,15,29	14,15,28
Nref	12067	11446
Tmin,Tmax	0.114,0.080	0.358,1.000
Tmin’	0.032	

Correction method= # Reported T Limits: Tmin=0.358 Tmax=1.000
AbsCorr = MULTI-SCAN

Data completeness= 0.949 Theta(max)= 78.057

R(reflections)= 0.0728(9525) wR2(reflections)= 0.1901(11446)

S = 1.046 Npar= 882

The following ALERTS were generated. Each ALERT has the format
test-name_ALERT_alert-type_alert-level.
Click on the hyperlinks for more details of the test.

Alert level C

```
PLAT213_ALERT_2_C Atom F00H           has ADP max/min Ratio .....       3.1 oblate
PLAT213_ALERT_2_C Atom F00J           has ADP max/min Ratio .....       3.5 prolat
PLAT213_ALERT_2_C Atom F01K           has ADP max/min Ratio .....       3.4 prolat
PLAT213_ALERT_2_C Atom F02L           has ADP max/min Ratio .....       3.7 prolat
PLAT213_ALERT_2_C Atom F02R           has ADP max/min Ratio .....       3.2 prolat
PLAT213_ALERT_2_C Atom C01P           has ADP max/min Ratio .....       3.4 oblate
PLAT213_ALERT_2_C Atom C028           has ADP max/min Ratio .....       3.4 prolat
PLAT213_ALERT_2_C Atom C02F           has ADP max/min Ratio .....       3.1 prolat
PLAT215_ALERT_3_C Disordered F36#     has ADP max/min Ratio .....       3.9 Note
PLAT220_ALERT_2_C NonSolvent   Resd 1  F   Ueq(max)/Ueq(min) Range       5.1 Ratio
PLAT230_ALERT_2_C Hirshfeld Test Diff for    F00F     --C02C     .       6.0 s.u.
PLAT234_ALERT_4_C Large Hirshfeld Difference F020     --C02B     .      0.19 Ang.
PLAT234_ALERT_4_C Large Hirshfeld Difference F02L     --C02P     .      0.20 Ang.
PLAT234_ALERT_4_C Large Hirshfeld Difference F02R     --C02P     .      0.20 Ang.
PLAT234_ALERT_4_C Large Hirshfeld Difference C02F     --C02P     .      0.17 Ang.
PLAT342_ALERT_3_C Low Bond Precision on  C-C Bonds ..............    0.0168 Ang.
PLAT431_ALERT_2_C Short Inter HL..A Contact  F01K      ..O00K     .      2.88 Ang.
                                                   x,-1+y,z  =      1_545 Check
PLAT906_ALERT_3_C Large K Value in the Analysis of Variance ......     5.292 Check
PLAT906_ALERT_3_C Large K Value in the Analysis of Variance ......     2.360 Check
PLAT911_ALERT_3_C Missing FCF Refl Between Thmin & STh/L=    0.600        69 Report
               -3  1  0,   3 11  0,   1 12  0,  -1 13  0,  -3 14  0,  -3-11  1,
               -3 -3  1,   9  5  1,   6  7  1,  -2 13  1,  -1 13  1,  -5  0  2,
                5  5  2,   9  5  2,   8  6  2,   6  7  2,   0 12  2,   1 12  2,
               -4 14  2,   8  6  3,   6  7  3,  -1 12  3,  -5 14  3,  -4 14  3,
               -3 -4  4,   8  6  4,  -1 12  4,  -4 13  4,  -3 13  4,  -5 14  4,
                   (   39 More Missing: see the .ckf listing file)
PLAT971_ALERT_2_C Check Calcd Resid. Dens.  1.04Ang From Ag05          1.88 eA-3
PLAT971_ALERT_2_C Check Calcd Resid. Dens.  1.05Ang From F02L          1.86 eA-3
PLAT971_ALERT_2_C Check Calcd Resid. Dens.  1.03Ang From Ag02          1.86 eA-3
PLAT971_ALERT_2_C Check Calcd Resid. Dens.  0.85Ang From F02L          1.85 eA-3
PLAT971_ALERT_2_C Check Calcd Resid. Dens.  1.07Ang From Ag04          1.79 eA-3
PLAT971_ALERT_2_C Check Calcd Resid. Dens.  1.06Ang From Ag03          1.76 eA-3
PLAT971_ALERT_2_C Check Calcd Resid. Dens.  1.10Ang From Ag01          1.71 eA-3
PLAT971_ALERT_2_C Check Calcd Resid. Dens.  1.17Ang From Ag01          1.69 eA-3
PLAT971_ALERT_2_C Check Calcd Resid. Dens.  0.95Ang From Ag01          1.67 eA-3
PLAT971_ALERT_2_C Check Calcd Resid. Dens.  1.26Ang From Ag05          1.65 eA-3
PLAT971_ALERT_2_C Check Calcd Resid. Dens.  1.08Ang From Ag06          1.60 eA-3
PLAT971_ALERT_2_C Check Calcd Resid. Dens.  1.09Ang From Ag06          1.60 eA-3
```

```
PLAT971_ALERT_2_C Check Calcd Resid. Dens.  1.15Ang From Ag04          1.59 eA-3
PLAT971_ALERT_2_C Check Calcd Resid. Dens.  0.80Ang From F02R          1.58 eA-3
PLAT971_ALERT_2_C Check Calcd Resid. Dens.  1.09Ang From Ag02          1.55 eA-3
PLAT971_ALERT_2_C Check Calcd Resid. Dens.  1.17Ang From Ag01          1.55 eA-3
PLAT971_ALERT_2_C Check Calcd Resid. Dens.  1.18Ang From Ag02          1.54 eA-3
PLAT971_ALERT_2_C Check Calcd Resid. Dens.  1.13Ang From F01G          1.52 eA-3
PLAT972_ALERT_2_C Check Calcd Resid. Dens.  0.81Ang From Ag01         -1.77 eA-3
PLAT972_ALERT_2_C Check Calcd Resid. Dens.  0.16Ang From F02L         -1.64 eA-3
PLAT972_ALERT_2_C Check Calcd Resid. Dens.  0.71Ang From Ag04         -1.58 eA-3
PLAT972_ALERT_2_C Check Calcd Resid. Dens.  0.10Ang From F02R         -1.53 eA-3
PLAT977_ALERT_2_C Check Negative Difference Density on H021      .    -0.52 eA-3
```

Alert level G

```
PLAT003_ALERT_2_G Number of Uiso or U(i,j)  Restrained non-H-Atoms        27 Report
PLAT042_ALERT_1_G Calc. and Reported MoietyFormula Strings  Differ     Please Check
              Calc: C38 H14 Ag6 F36 N12 O4
              Rep.: 2(C15 H3 Ag3 F18 N6), C8 H8 O4
PLAT083_ALERT_2_G SHELXL Second Parameter in WGHT  Unusually Large      75.19 Why ?
PLAT154_ALERT_1_G The s.u.'s on the Cell Angles are Equal ..(Note)      0.002 Degree
PLAT171_ALERT_4_G The CIF-Embedded .res File Contains EADP Records         2 Report
PLAT177_ALERT_4_G The CIF-Embedded .res File Contains DELU Records         3 Report
PLAT178_ALERT_4_G The CIF-Embedded .res File Contains SIMU Records         4 Report
PLAT186_ALERT_4_G The CIF-Embedded .res File Contains ISOR Records         7 Report
PLAT242_ALERT_2_G Low    'MainMol' Ueq as Compared to Neighbors of      C01M Check
PLAT242_ALERT_2_G Low    'MainMol' Ueq as Compared to Neighbors of      C01X Check
PLAT242_ALERT_2_G Low    'MainMol' Ueq as Compared to Neighbors of      C01Z Check
PLAT242_ALERT_2_G Low    'MainMol' Ueq as Compared to Neighbors of      C025 Check
PLAT242_ALERT_2_G Low    'MainMol' Ueq as Compared to Neighbors of      C026 Check
PLAT242_ALERT_2_G Low    'MainMol' Ueq as Compared to Neighbors of      C02B Check
PLAT242_ALERT_2_G Low    'MainMol' Ueq as Compared to Neighbors of      C02E Check
PLAT242_ALERT_2_G Low    'MainMol' Ueq as Compared to Neighbors of      C02H Check
PLAT242_ALERT_2_G Low    'MainMol' Ueq as Compared to Neighbors of      C02K Check
PLAT242_ALERT_2_G Low    'MainMol' Ueq as Compared to Neighbors of      C02N Check
PLAT242_ALERT_2_G Low    'MainMol' Ueq as Compared to Neighbors of      C02P Check
PLAT299_ALERT_4_G Atom Site Occupancy Constrained at ............        0.5 Check
              F2      F19     F20     F027    F02P    F01D
PLAT301_ALERT_3_G Main Residue  Disorder .............(Resd    1)         3% Note
PLAT333_ALERT_2_G Large Aver C6-Ring C-C Dist C01E     -C024     .       1.44 Ang.
PLAT335_ALERT_2_G Check Large C6 Ring C-C Range C01E     -C024           0.17 Ang.
PLAT434_ALERT_2_G Short Inter HL..HL Contact F00T     ..F02R     .       2.81 Ang.
                                                  x,-1+y,z  =      1_545 Check
PLAT434_ALERT_2_G Short Inter HL..HL Contact F01A     ..F020     .       2.83 Ang.
                                                   x,1+y,z  =      1_565 Check
PLAT605_ALERT_4_G Largest Solvent Accessible VOID in the Structure        142 A**3
PLAT720_ALERT_4_G Number of Unusual/Non-Standard Labels ..........        110 Note
              Ag01    Ag02    Ag03    Ag04    Ag05    Ag06    F007    F008
              F009    F00A    F00B    F00C    O00D    F00E    F00F    F00G
              F00H    F00I    F00J    O00K    F00L    F00M    F00N    N00O
              O00P    F00Q    N00R    F00S    F00T    F00U    O00W    N00X
              F00Y    N00Z    N010    F011    F012    N013    F014    N015
              N016    N017    N018    F019    F01A    N01B    C01D    H01D
              C01E    H01E    F01F    F01G    N01H    C01I    C01J    F01K
              C01L    C01M    F01N    C01O    H01A    H01B    H01C    C01P
              H01P    C01Q    C01R    C01S    C01T    H01T    C01U    C01V
              C01W    C01X    C01Y    H01Y    C01Z    F020    C021    H021
              C022    H022    C023    C024    C025    C026    F027    C028
```

```
              C029    F02A    C02B    C02C    C02D    C02E    C02F    C02G
              H02G    C02H    C02I    C02J    H02A    H02B    H02C    C02K
              F02L    C02N    C02P    F02R    F02P    F01D
PLAT860_ALERT_3_G Number of Least-Squares Restraints ............        198 Note
PLAT868_ALERT_4_G ALERTS Due to the Use of _smtbx_masks Suppressed         ! Info
PLAT883_ALERT_1_G Absent Datum for _atom_sites_solution_primary ..     Please Do !
PLAT910_ALERT_3_G Missing FCF Reflection(s) Below Theta(Min)[Deg]=      3.87 Note
                0  0  1,
PLAT912_ALERT_4_G Missing # of FCF Reflections Above STh/L=  0.600        550 Note
PLAT913_ALERT_3_G Missing # of Very Strong Reflections in FCF ....          1 Note
               -3  1  0,
PLAT933_ALERT_2_G Number of HKL-OMIT Records in Embedded .res File         15 Note
               -2 13  1,  -1 11  6,  -1 12  3,  -1 12  4,  -1 13  0,  -1 13  1,
                0 11  6,   0 12  2,   1 12  0,   1 12  2,   3  9 10,   3  9 11,
                6  7  1,   6  7  2,   6  7  3,
PLAT941_ALERT_3_G Average HKL Measurement Multiplicity ..........        2.4 Low
PLAT969_ALERT_5_G The 'Henn et al.' R-Factor-gap value ..........      3.838 Note
              Predicted wR2: Based on SigI**2  4.95 or SHELX Weight 18.18
PLAT978_ALERT_2_G Number C-C Bonds with Positive Residual Density.          0 Info
```

```
   0 ALERT level A = Most likely a serious problem - resolve or explain
   0 ALERT level B = A potentially serious problem, consider carefully
  43 ALERT level C = Check. Ensure it is not caused by an omission or oversight
  37 ALERT level G = General information/check it is not something unexpected

   3 ALERT type 1 CIF construction/syntax error, inconsistent or missing data
  53 ALERT type 2 Indicator that the structure model may be wrong or deficient
  10 ALERT type 3 Indicator that the structure quality may be low
  13 ALERT type 4 Improvement, methodology, query or suggestion
   1 ALERT type 5 Informative message, check
```

Validation response form

Please find below a validation response form (VRF) that can be filled in and pasted into your CIF.

```
# start Validation Reply Form
_vrf_PLAT213_lcz003
;
PROBLEM: Atom F00H            has ADP max/min Ratio .....        3.1 oblate
RESPONSE: ...
;
_vrf_PLAT215_lcz003
;
PROBLEM: Disordered F36#      has ADP max/min Ratio .....        3.9 Note
RESPONSE: ...
;
_vrf_PLAT220_lcz003
;
PROBLEM: NonSolvent   Resd 1  F   Ueq(max)/Ueq(min) Range        5.1 Ratio
RESPONSE: ...
;
_vrf_PLAT230_lcz003
;
PROBLEM: Hirshfeld Test Diff for    F00F     --C02C     .        6.0 s.u.
RESPONSE: ...
```

```
;
_vrf_PLAT234_lcz003
;
PROBLEM: Large Hirshfeld Difference F020     --C02B     .       0.19 Ang.
RESPONSE: ...
;
_vrf_PLAT342_lcz003
;
PROBLEM: Low Bond Precision on  C-C Bonds ..............     0.0168 Ang.
RESPONSE: ...
;
_vrf_PLAT431_lcz003
;
PROBLEM: Short Inter HL..A Contact  F01K     ..O00K     .       2.88 Ang.
RESPONSE: ...
;
_vrf_PLAT906_lcz003
;
PROBLEM: Large K Value in the Analysis of Variance ......      5.292 Check
RESPONSE: ...
;
_vrf_PLAT911_lcz003
;
PROBLEM: Missing FCF Refl Between Thmin & STh/L=    0.600         69 Report
RESPONSE: ...
;
_vrf_PLAT971_lcz003
;
PROBLEM: Check Calcd Resid. Dens.  1.04Ang From Ag05           1.88 eA-3
RESPONSE: ...
;
_vrf_PLAT972_lcz003
;
PROBLEM: Check Calcd Resid. Dens.  0.81Ang From Ag01          -1.77 eA-3
RESPONSE: ...
;
_vrf_PLAT977_lcz003
;
PROBLEM: Check Negative Difference Density on H021      .      -0.52 eA-3
RESPONSE: ...
;
# end Validation Reply Form
```

It is advisable to attempt to resolve as many as possible of the alerts in all categories. Often the minor alerts point to easily fixed oversights, errors and omissions in your CIF or refinement strategy, so attention to these fine details can be worthwhile. It is up to the individual to critically assess their own results and, if necessary, seek expert advice.

PLATON version of 17/09/2025; check.def file version of 17/09/2025

---

duplicate check

No duplication found

Datablock lcz003 - ellipsoid plot

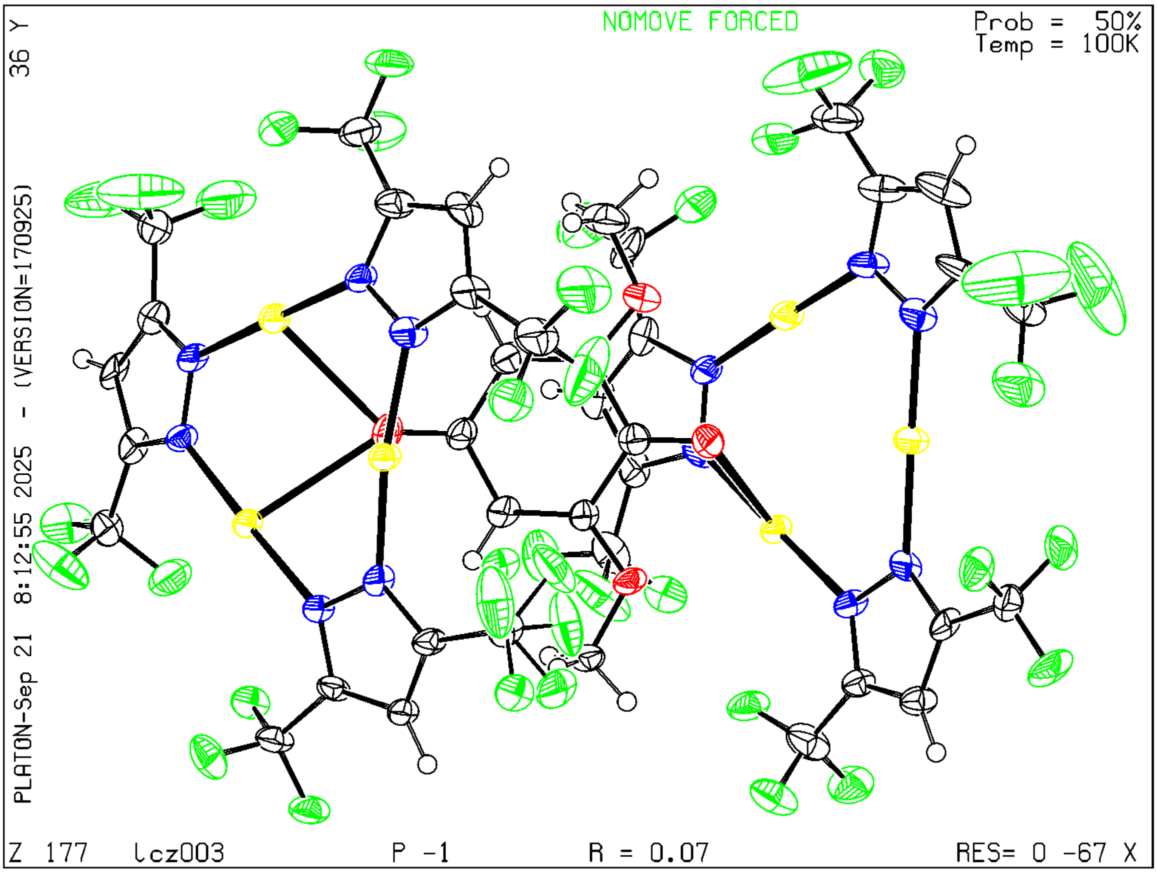

Supplement: Supplementary file 2 — Supporting File 2: anie72462‐sup‐0002‐DataFile.zip. [file ANIE-65-e1218503-s002.zip › supplementary_materials_crystallographic_data submit.zip!/cif_and_checkcif(1-114)/Ag3Pz3_3/Ag3Pz3_3_checkcif.pdf]
